# Relevance of *HOTAIR* rs920778 and rs12826786 Genetic Variants in Bladder Cancer Risk and Survival

**DOI:** 10.3390/cancers16020434

**Published:** 2024-01-19

**Authors:** Eduarda P. Martins, Joana Vieira de Castro, Rita Fontes, Sara Monteiro-Reis, Rui Henrique, Carmen Jerónimo, Bruno M. Costa

**Affiliations:** 1 Life and Health Sciences Research Institute (ICVS), School of Medicine, Campus de Gualtar, University of Minho, 4710-057 Braga, Portugal; eduardamartins@med.uminho.pt (E.P.M.); joana.vieira.castro@gmail.com (J.V.d.C.); aritacfontes@gmail.com (R.F.); 2 ICVS/3B’s-PT Government Associate Laboratory, 4710-057/4805-017 Braga/Guimarães, Portugal; 3 Cancer Biology & Epigenetics Group, Research Center of IPO Porto (CI-IPOP), CI-IPOP @RISE (Health Research Network), Portuguese Oncology Institute of Porto (IPO Porto), Porto Comprehensive Cancer Center Raquel Seruca (Porto.CCC), 4200-072 Porto, Portugal; sara.raquel.reis@ipoporto.min-saude.pt (S.M.-R.); henrique@ipoporto.min-saude.pt (R.H.); carmenjeronimo@ipoporto.min-saude.pt (C.J.); 4 Institute of Science and Innovation in Mechanical and Industrial Engineering (INEGI), University of Porto, 4200-465 Porto, Portugal; 5 Department of Pathology & Molecular Immunology, ICBAS-School of Medicine & Biomedical Sciences, University of Porto, 4050-313 Porto, Portugal; 6 Department of Pathology, Portuguese Oncology Institute of Porto (IPO Porto), 4200-072 Porto, Portugal

**Keywords:** *HOTAIR*, rs920778, rs12826786, SNP, bladder cancer

## Abstract

**Simple Summary:**

Bladder cancer is the 10th most diagnosed cancer worldwide. The long non-coding RNA HOX transcript antisense intergenic RNA (*HOTAIR*) has been shown to play pivotal oncogenic roles in this type of cancer. The aim of this study was to determine the relevance of the *HOTAIR* rs920778 and rs12826786 genetic variants in bladder cancer susceptibility and prognosis. Our retrospective analyses using 106 bladder cancer patients and 199 cancer-free controls demonstrated that, despite not presenting an association with bladder cancer risk, *HOTAIR* rs920778 TT and rs12826786 CC genotypes are associated with a better prognosis for bladder cancer patients.

**Abstract:**

The long non-coding RNA HOX transcript antisense intergenic RNA (*HOTAIR*) is associated with oncogenic features in bladder cancer and is predictive of poor clinical outcomes in patients diagnosed with this disease. In this study, we evaluated the impact of the *HOTAIR* single nucleotide polymorphisms rs920778 and rs12826786 on bladder cancer risk and survival. This case-control study included 106 bladder cancer patients and 199 cancer-free controls. Polymorphisms were evaluated through PCR-restriction fragment length polymorphism. The odds ratio and 95% confidence intervals were tested using univariable and multivariable logistic regressions. The effects on patient survival were evaluated using the log-rank test and Cox regression models. Our data showed that the *HOTAIR* rs920778 and rs12826786 genetic variants are not associated with the risk of developing bladder cancer. Nevertheless, survival analyses suggested that the *HOTAIR* rs920778 TT genotype and rs12826786 CC genotype are associated with increased survival in male bladder cancer patients and in patients, both male and female, who have primary tumors with a pathological stage of pT2. Together, these results suggest that, despite not being associated with bladder cancer risk, *HOTAIR* rs920778 and rs12826786 polymorphisms might represent new prognostic factors in this type of cancer. This is particularly important as these polymorphisms might be easily evaluated in bladder cancer patients in a minimally invasive manner to better predict their clinical outcomes.

## 1. Introduction

Bladder cancer ranks as the 10th most common form of cancer worldwide, accounting for >573,000 new estimated cases and >212,000 deaths in 2020 [1]. This type of tumor is more frequent among white males and older individuals, as it is the sixth most incident cancer and accounts for 2.9% of cancer-related deaths in males [1]. The treatment of patients diagnosed with bladder cancer varies according to the disease’s aggressiveness: non-muscle invasive tumors are usually treated with transurethral resection of the bladder tumor, which may be followed by intravesical immunotherapy or chemotherapy administration, taking into consideration the risk of progression [2,3]; the more aggressive forms—muscle-invasive tumors—are treated with radical cystectomy and cisplatin-based neoadjuvant chemotherapy [2,4]. Patient survival significantly differs based on the stage of the disease. For instance, those with low-grade Ta tumors exhibit a 15-year progression-free survival of 95%, while individuals diagnosed with metastatic bladder cancer have a median survival of only ~12 months [5]. In contrast to other cancer types for which the main risk factors remain undisclosed, bladder cancer susceptibility is known to be associated with smoking habits, consumption of water containing arsenic, and occupational exposure to carcinogens including aromatic amines [6,7,8]. More recent studies also suggest that particular dietary factors, including low hydration; low intake of vitamins A, D, and folate; and consumption of animal protein and processed meat, are putative or suspected risk factors [8]. Considering the devastating rates of incidence and mortality associated with bladder cancer worldwide, many efforts have been made to identify new biomarkers and develop new minimally invasive methods for diagnosis and follow-up [9,10,11].

The long non-coding RNA (lncRNA) HOX transcript antisense intergenic RNA (*HOTAIR*), first described in 2007, was initially suggested as being implicated in key molecular processes, including chromatin regulation [12]. This ability to reprogram chromatin was quickly linked to cancer [13], and today, *HOTAIR* is recognized as relevant in various tumor types [13,14,15,16,17,18,19,20,21,22]. In bladder cancer, this lncRNA has consistently been identified as an oncogenic factor, influencing in vitro functional capacities such as invasion and migration [23,24] and being associated with poor prognoses in bladder cancer patients [25,26,27,28,29]. Specifically, *HOTAIR* is upregulated in bladder cancer samples as compared to non-tumoral tissues [26,29,30], and this high expression is predictive of a shorter overall survival, time-to-recurrence, and disease progression [24,25,26,27,28,29]. Beyond its prognostic significance, *HOTAIR* might also hold diagnostic importance in bladder cancer, as it is enriched in exosomes isolated from the urine samples of patients diagnosed with muscle-invasive high-grade urothelial bladder cancer [23].

Despite numerous studies exploring the functional and prognostic relevance of *HOTAIR* in bladder cancer, research on *HOTAIR* single-nucleotide polymorphisms (SNPs) remains limited. A few studies have primarily focused on six loci (rs4759314, rs7958904, rs874945, rs920778, rs1899663, and rs12427129) [25,31]. These studies associate the rs874945 AG/AA genotype with an increased risk of bladder cancer as compared to the GG genotype [31]; the rs920778 C allele is negatively correlated with lymph node metastases [25], the rs4759314 AG+GC in female patients is linked to an increased risk of urothelial cell carcinoma and shorter survival [25], and the T allele of rs12427129 in smokers or younger patients is associated with a higher risk of advanced tumors [25].

Thus, given the limited knowledge regarding *HOTAIR* SNPs in bladder cancer, this study aims to assess the impact of rs920778 (C > T) and rs12826786 (C > T), which are described as potentially affecting *HOTAIR* expression [32,33,34,35,36] and are indicated as two of the most promising *HOTAIR* SNPs to be used as genetic biomarkers in cancer [37,38], in the risk and prognosis of bladder cancer patients. These studies are particularly relevant as they might help in the identification of new biomarkers for recognizing individuals with higher susceptibilities to developing bladder cancer, which would impact cancer prevention and screening policies. In addition, the evaluated genetic variants might also present a prognostic value and be used to better predict the clinical outcome of bladder cancer patients using minimally invasive methodologies.

## 2. Materials and Methods

### 2.1. Study Population

This study included 106 DNA samples from peripheral blood obtained from bladder cancer patients diagnosed at the Portuguese Oncology Institute of Porto (IPO Porto, Portugal), between 1993 and 2011, all whom had complete follow-up information. As controls, adult cancer-free individuals without any reference to chronic disease or medication were randomly selected from blood donors (*n* = 199). The control group had previously been characterized for *HOTAIR* rs920778 and rs12826786 polymorphisms [35]. All subjects included in the study were from Caucasian ethnic backgrounds. Tumors were classified based on their histopathological features and were categorized according to their pathological stages (pTNM) [39] and grades. The clinicopathological data of patients and the information on the controls are summarized in Table 1. This study was approved by the respective ethical entity (CES IPOPFG-EPE 019/08), and all subjects provided written informed consent.

### 2.2. Single Nucleotide Polymorphism Genotyping

Genomic DNA was extracted from the peripheral blood leukocytes using a proteinase K/phenol-chloroform/ethanol treatment. The samples were digested in 10% sodium dodecyl sulfate and proteinase K (20 mg/mL) overnight at 55 °C. The *HOTAIR* polymorphisms rs920778 (C > T) and rs12826786 (C > T) were genotyped by PCR-restriction fragment length polymorphism (RFLP) method. Briefly, PCR amplification was performed with 50 ng of DNA and KAPA Taq DNA Polymerase (KAPA Biosystems, Wilmington, MA, USA). Primers sequences were previously described [35,40]: for the rs920778 polymorphism, the sense primer was 5′- TTACAGCTTAAATGTCTGAATGTTCC-3′, and the antisense primer was 5′-TATGCGCTTTGCTTCCAGTT-3′; for the rs12826786 polymorphism, the sense primer was 5′-GGGCTGGTTTAGATTGGCT-3′, and the antisense primer was 5′-GAGCGGCTGGAGTCTGAGG-3′. For the rs920778 polymorphism, PCR conditions were as follows: DNA denaturation at 95 °C for 5 min, 40 cycles of 95 °C for 30 s, 56 °C for 30 s and 72 °C for 30 s, finishing with a final extension cycle at 72 °C for 8 min. For rs12826786 polymorphism, a touchdown PCR was conducted: after DNA denaturation, the reaction occurred for nine cycles at 95 °C for 30 s, 68–64 °C for 30 s (lowering ~0.5 °C/cycle) and 72 °C for 30 s, followed by 26 cycles of 95 °C for 30 s, 64 °C for 30 s, and 72 °C for 30 s, and a final extension cycle at 72 °C for 8 min. The PCR products were digested at 37 °C for 5 min (rs920778) with Fast Digest MspI (Thermo Scientific, Waltham, MA, USA) or for 30 min (rs12826786) with Fast Digest BgIII (Thermo Scientific, Waltham, MA, USA). Digestion products were resolved in 4% agarose gel and detected using GreenSafe Premium (NZYTech, Lisbon, Portugal).

### 2.3. Statistical Analyses

The differences in the frequencies of genotypes and alleles between the control and patients groups were determined using the Chi-square test. The linkage disequilibrium of both SNPs in our datasets was calculated using the SHEsis program [41]. The odds ratio (OR) with 95% confidence intervals for univariable and multivariable analyses was determined by logistic regression. Univariable survival analyses were plotted by Kaplan-Meier survival curves, and the effects of the genotypes were assessed with the log-rank test. Multivariable survival analyses were performed through the Cox regression model and were adjusted for patients’ ages at diagnosis (as a continuous variable), sex, and primary tumor classifications when applicable. Overall survival was defined as the time from diagnosis to death, and recurrence-free survival was defined as the time from diagnosis to local/regional recurrence or death. Statistical tests were not performed in groups of patients with a sample size of less than 20. Analyses were performed using IBM SPSS Statistics (Armonk, NY, USA, version 27) software. Statistically significant differences were considered when the *p*-values were lower than 0.05.

## 3. Results

### 3.1. Population Characteristics

This case-control study involved 106 bladder cancer patients and 199 cancer-free individuals. Bladder cancer patients presented a mean age at diagnosis of 67.35 years (ranging between 37 and 91 years), with 83 males and 23 females (a ratio of 3.6:1), reflecting the higher frequency of diagnoses in male patients [1]. The control group included 130 males and 69 females, with a mean age of 46.42 (ranging between 27 and 85 years). Further details regarding bladder cancer patients, such as primary tumor stage classifications and grades can be found in Table 1.

### 3.2. Genotype and Allelic Distributions of HOTAIR SNPs

The frequencies of TT, CC, and CT for *HOTAIR* rs920778 were 45.2, 12.6, and 42.2% for the cancer-free individuals, and 46.2, 13.2, and 40.6% for the bladder cancer patients (Table 2). The T allele was the most frequent in both groups (66.3% and 66.5% in the controls and bladder cancer patients, respectively). For *HOTAIR* rs12826786, the frequencies of TT, CC, and CT were 10.6, 47.2, and 42.2% for the controls, and 5.7, 49.1, and 45.3% for the bladder cancer patients. The C allele was the most frequent (68.3% in the cancer-free individuals and 71.7% in the bladder cancer samples, Table 2). The distribution of genotypic and allelic frequencies of both SNPs did not show statistically significant differences between the two groups (*p* > 0.05), and the distribution of both polymorphisms in cancer-free individuals was in Hardy-Weinberg equilibrium (*p* = 0.750 for rs920778 and *p* = 0.935 for rs12826786), as previously reported [35]. Moreover, the *HOTAIR* rs920778 and rs12826786 SNPs were in strong linkage disequilibrium (D’ = 0.93 and *r*^2^ = 0.75). Importantly, the Pearson Chi-square tests did not reveal statistically significant differences in the distribution of TT, CC, and CT genotypes for either polymorphism between males and females when tested in all subjects, as well as in the controls and bladder cancer cases separately. The same observations were made regarding the alleles (T and C) for the two *HOTAIR* polymorphisms.

### 3.3. HOTAIR rs920778 and rs12826786 SNPs and Bladder Cancer Risk

Initially, we conducted univariable analyses to examine whether specific variants of these *HOTAIR* polymorphisms were associated with bladder cancer risk (Table 2). Focusing on *HOTAIR* rs920778, we observed that individual alleles were not associated with bladder cancer risk. When using the TT genotype as a reference (the most prevalent genotype in the control group), we found that the CC, CT, and combined CC+CT genotypes were also not associated with bladder cancer risk.

Similarly, concerning *HOTAIR* rs12826786, with the C allele and CC genotype considered as the references due to their higher representations in the control group, the T allele, as well as the TT, CT, and combined TT+CT genotypes, were not associated with bladder cancer risk. The association between both *HOTAIR* SNPs and bladder cancer susceptibility was further examined through a multivariable analysis, adjusting for sex and age (as continuous variable, Table 3). As expected, increased age was associated with an increased risk of developing bladder cancer, while women presented decreased susceptibility to these tumors, which is consistent with the previous literature [42]. However, in line with the results from the univariable analyses, the *HOTAIR* rs920778 and rs12826786 SNPs did not show an association with bladder cancer risk. Univariable and multivariable analyses were also performed in specific patient groups individually (pT0-1/pT2-4; individual pT groups and grades; male/female; and younger/older than 65 years old), and again, no significant associations were found between the different genotypes and bladder cancer risk. Finally, univariable and multivariable risk analyses were repeated using a selected subgroup of controls (*n* = 93; Supplementary Table S1) with a considerably more similar age interval to bladder cancer cases, which validated the same lack of significant associations (Supplementary Tables S2 and S3).

### 3.4. Effects of HOTAIR rs920778 and rs12826786 on Prognosis of Bladder Cancer Patients

**Figure 1:**
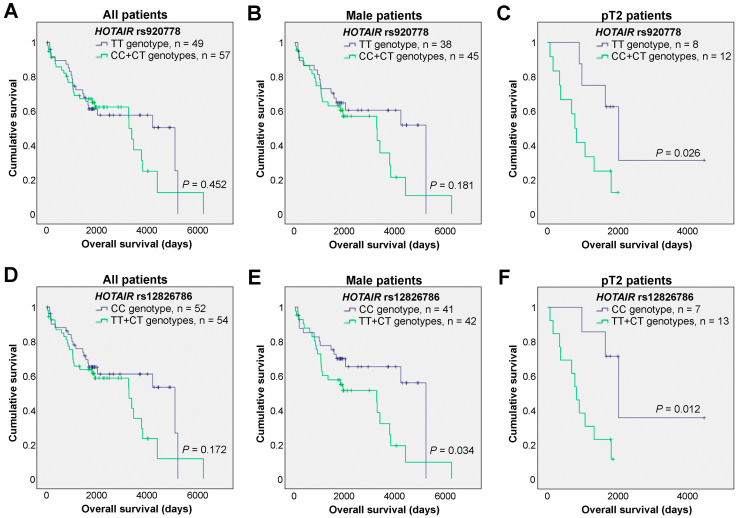
Effects of the *HOTAIR* rs920778 and rs12826786 polymorphisms in the overall survival of bladder cancer patients. (**A**–**C**) Kaplan-Meier survival curves of all bladder cancer patients (**A**), male patients (**B**), and patients with tumors staged as pT2 (**C**) according to the *HOTAIR* rs920778 genotypes. (**D**–**F**) Kaplan-Meier survival curves of all bladder cancer patients (**D**), male patients (**E**), and patients with tumors staged as pT2 (**F**) according to the *HOTAIR* rs12826786 genotypes (Log-rank tests were applied; tick marks represent censored data).

To assess whether the *HOTAIR* rs920778 and rs12826786 genetic variants could impact the survival of diagnosed bladder cancer patients, univariable analyses were initially performed. No statistically significant associations of the distinct genotypes were observed in the overall survival of bladder cancer patients for both polymorphisms (Figure 1A,D).

Considering the association of bladder cancer with patient sex, particularly its higher frequency in men which was also evident in our cohort, we decided to narrow our focus to male patients (*n* = 83). Interestingly, while the *HOTAIR* rs920778 genotypes did not significantly associate with overall survival (Figure 1B), in the case of *HOTAIR* rs12826786, patients harboring the CC genotype demonstrated a significantly better prognosis compared to all others (TT+CT genotypes, Figure 1E). The effect of the two *HOTAIR* SNPs was then tested in patients who were stratified according to their primary tumor pathological stages. Here, a statistically significant effect of each of the SNPs was identified in the overall survival of the pT2 patients (*n* = 20). Specifically, for *HOTAIR* rs920778, pT2 patients with TT genotypes exhibited increased overall survival as compared to all other patients (Figure 1C). Additionally, for *HOTAIR* rs12826786 genetic variants, the pT2 patients with the CC genotype showed longer overall survival than the remaining patients (Figure 1F).

Critically, these results were further investigated at the multivariable level, taking into consideration clinically important variables such as patient age, sex, and primary tumor pathological stage (Table 4). Similar to the univariable analyses, when examining the impact of both *HOTAIR* polymorphisms in all patients included in the dataset, no differences were observed. Nevertheless, when focusing on male patients, as well as patients with pT2 tumors, both *HOTAIR* SNPs showed significant associations with prognoses. Specifically, for *HOTAIR* rs920778 in male patients, those patients with combined CC and CT genotypes exhibited an increased hazard ratio as compared to TT patients (Table 4), which is in line with the tendency observed in the univariable analysis where TT patients showed a slight increase in survival (Figure 1B). For pT2 patients, the patients carrying the TT genotype presented a significant increased survival as compared to the CT patients, which was further consolidated when compared to all other patients (CT+CC). Regarding the *HOTAIR* rs12826786 genetic variants, the CC genotype consistently showed an association with longer overall survival when compared to CT and combined TT+CT patients in the male subgroup. Similarly, in the subgroup of patients with pT2 tumors, the CC genotype was associated with a better prognosis when compared to all other patients (individual TT, CT, and combined TT+CT genotypes). We also tested the effects on the survival of patients with tumor stages pT2-pT4 (*n* = 33), and, in this larger but more heterogeneous group of patients, *HOTAIR* rs12826786 still maintained its prognostic value (CT vs. CC: HR = 2.865 [1.026–7.999], *p* = 0.044; TT+CT vs. CC: HR = 2.746 [1.046–7.210], *p* = 0.040; Supplementary Table S4).

The influence of the *HOTAIR* rs920778 and rs12826786 SNPs on recurrence-free survival was further explored. While no major differences were observed for all patients in this dataset and in particular subgroups of patients when tested at the univariable level, multivariable analyses in older patients (≥65 years, both sexes; *n* = 67) showed that those carrying the CC genotype in rs12826786 demonstrated a significantly prolonged recurrence-free survival as compared to individuals with the TT genotype and all other patients (Supplementary Table S5).

These results suggest that, while lacking prognostic value in the whole and heterogeneous population of bladder cancer patients, both the *HOTAIR* rs920778 and rs12826786 genetic variants exhibit significant implications for the outcomes of specific subgroups of patients, stratified according to clinically relevant variables like age, sex, and tumor stages.

## 4. Discussion

This case-control study, comprising 106 Portuguese bladder cancer patients and 199 cancer-free control individuals, focused on exploiting the relevance of two genetic variants of *HOTAIR*—an acknowledged oncogenic molecule in bladder cancer [23,24,26,29]—in the risk and prognosis of bladder cancer patients. The study of SNPs in cancer is a promising strategy, as SNPs can potentially serve as biomarkers for diagnosis or prognosis. Notably, SNPs offer advantages which include the ability to be easily tested through non-invasive approaches in patients’ peripheral blood [43]. Indeed, the relevance of non-coding RNAs, both at the levels of SNPs and alterations in gene expression levels, has been widely studied in cancer and proposed as being useful blood-based biomarkers [44,45,46]. While certain polymorphisms in NAT and GST genes with established associations with bladder cancer susceptibility have been well-studied (reviewed in [47]), understanding the complexity associated with bladder cancer risk and prognosis requires a better understanding of other SNPs in distinct populations.

In this study we focused on *HOTAIR*, not only for its known relevance in bladder cancer [23,24,26,29], but also for its critical role in other cancer types. Specifically, *HOTAIR* has been identified as a determinant factor in the response of cancer to therapy; in invasion, migration, and proliferation; and in maintaining stemness features and other functional effects, primarily through interactions with other molecules including microRNAs [19,21,48]. Importantly, these functional and mechanistic effects have been shown to be accompanied by clinically relevant implications, as the overexpression of *HOTAIR* is linked to a dismal prognosis in patients diagnosed with numerous types of cancer (e.g., breast cancer [49], esophageal squamous cell carcinoma [50,51], colorectal cancer [22], glioblastoma [14], and others) [15].

Through both univariable and multivariable logistic analyses, our findings suggest no association between *HOTAIR* rs920778 and rs12826786 SNPs and bladder cancer risk in all tested groups. To the best of our knowledge, this is the first study focused on understanding the relevance of the *HOTAIR* rs920778 and rs12826786 genetic variants in bladder cancer susceptibility within a Caucasian population. Importantly, our data fit well with the data previously reported by Tung et al., indicating that rs920778 SNPs were not associated with urothelial cell carcinoma risk in patients of Asian background [25].

Considering that the high expression of *HOTAIR* is a predictor of shorter survival in bladder cancer patients [24,25,26,27,28,29], we also explored the potential effects of the *HOTAIR* rs920778 and rs12826786 SNPs on survival. Our findings reveal that specific groups of patients (male patients and pT2 bladder cancer patients) carrying the TT genotype in *HOTAIR* rs920778 have significantly longer overall survival. Similarly, within these patient groups, those with the CC genotype of *HOTAIR* rs12826786 present had increased survival as compared to those with the TT or CT genotypes.

The TT genotype in *HOTAIR* rs920778 has been associated with increased *HOTAIR* expression in esophageal normal tissue [32], cervical cancer and normal tissues [36], and gastric cancer and normal tissues [34]. Considering that *HOTAIR* acts mostly as an oncogene in bladder cancer, and that, in our case-control study, we found that the TT genotype is associated with a better prognosis, it would be of paramount importance to understand if, in this specific tumor type, the TT genotype is also correlated with an increased expression of *HOTAIR*. Importantly, the existing evidence indicates no association between the *HOTAIR* rs920778 genotypes and *HOTAIR* expression in glioma patients [35], which strengthens the notion that, according to the tumor type or population background, the impact of a certain genotype in gene expression or in cancer susceptibility might differ. Notably, all the studies mentioned above reporting the rs920778 TT genotype’s association with an increased expression of *HOTAIR* were based on Asian populations. Furthermore, a study published in 2016 highlighted that the T allele of *HOTAIR* rs920778 was significantly associated with an increased risk of developing cancer in Asians but not in a Turkish population [52], emphasizing the importance of ethnicity in this type of studies. In addition to the need for validating our results in larger and more diverse populations with distinct ethnicities, it will also be crucial to consider the exposure to known environmental factors linked to bladder cancer, including smoking, workplace chemical exposures (e.g., aromatic amines), arsenic in drinking water, and others. It is particularly interesting to note that while the *HOTAIR* rs920778 and rs12826786 genetic variants seem not to affect the patient’s susceptibility to developing bladder cancer, they might influence patient outcomes. This suggests that the functional effect of these SNPs might be more clinically relevant once the disease is established.

Our study adds to the body of data implicating particular non-coding RNAs, including both lncRNAs and microRNAs, as key molecules in the context of bladder cancer [53]. For example, in the last decade, urinary micro-RNAs have emerged as promising tools to be explored in the context of liquid biopsy [54]. Some examples include the overexpression of miR-126, miR-182, and miR-199a in the urine of bladder cancer patients, which suggests a diagnostic capacity [54,55]. The quest for new biomarkers has also been recently extended to the urinary microbiome field (urobiome) [56,57], as some types of bacteria might contribute to tumor-promoting inflammation. Interestingly, a recent case-control study identified an accumulation of *Porphyromonas* and *Porphyromonas somerae* in the first morning urine of bladder cancer patients, suggesting that these bacteria present the potential to be used as biomarkers for identifying individuals with higher likelihoods of presenting bladder cancer [57]. The integration of these multiple findings might contribute to developing more refined bladder-cancer-risk stratification tools based on multiple biomarkers from genetic factors to environmental exposures and microbiota.

## 5. Conclusions

Our data suggest that the *HOTAIR* rs920778 and rs12826786 polymorphisms might be independent prognostic factors in specific subgroups of bladder cancer patients, namely, in male patients or in patients with a pathological tumor stage of pT2. Considering the limited sample size and the prognostic effects restricted to particular patients’ subgroups, future studies are warranted in order to validate these findings in larger and ethnically diverse cohorts of patients.

## Figures and Tables

**Table 1:** Clinicopathological information of bladder cancer patients (cases) and cancer-free individuals (controls).

	Bladder Cancer Cases	Controls
Number	106	199
Age, mean [range]	67.35 [37–91]	46.42 [27–85]
Sex, n (%)		
Male	83 (78.3%)	130 (65.3%)
Female	23 (21.7%)	69 (34.7%)
Grade, n (%)		–
PLG	49 (46.2%)	
PHG	30 (28.3%)	
IHG	27 (25.5.%)	
Pathological Stage (pT), n (%)		–
pTis	4 (3.8%)	
pTa	47 (44.3%)	
pT1	22 (20.8%)	
pT2	20 (18.9%)	
pT3	9 (8.5%)	
pT4	4 (3.8%)	

PLG: Papillary Low Grade; PHG: Papillary High Grade; IHG: Invasive High Grade.

**Table 2:** Univariable logistic regression analysis of the association between the *HOTAIR* rs920778 and rs12826786 genetic variants and bladder cancer risk.

Polymorphism	Controls	Cases	OR [95% CI] ^a^	*p*-Value
***HOTAIR* rs920778**				
Genotype				
TT	90 (45.2%)	49 (46.2%)	-	0.960
CT	84 (42.2%)	43 (40.6%)	0.940 [0.567–1.560]	0.811
CC	25 (12.6%)	14 (13.2%)	1.029 [0.490–2.158]	0.941
CC+CT	109 (54.8%)	57 (53.8%)	0.960 [0.599–1.541]	0.867
Alleles				
T	264 (66.3%)	141 (66.5%)	-	-
C	134 (33.7%)	71 (33.5%)	0.992 [0.697–1.412]	0.965
***HOTAIR* rs12826786**				
Genotype				
CC	94 (47.2%)	52 (49.1%)	-	0.367
CT	84 (42.2%)	48 (45.3%)	1.033 [0.633–1.687]	0.897
TT	21 (10.6%)	6 (5.7%)	0.516 [0.196–1.360]	0.181
TT+CT	105 (52.8%)	54 (50.9%)	0.930 [0.580–1.490]	0.762
Alleles				
C	272 (68.3%)	152 (71.7%)	-	-
T	126 (31.7%)	60 (28.3%)	0.852 [0.591–1.229]	0.391

^a^ Odds ratio (OR) with 95% confidence intervals (CI).

**Table 3:** Multivariable logistic regression analysis of the association between the *HOTAIR* rs920778 and rs12826786 genetic variants and bladder cancer risk.

Polymorphism	Controls	Cases	OR [95% CI] ^a^	*p*-Value
***HOTAIR* rs920778**				
Genotype				
TT	90 (45.2%)	49 (46.2%)	-	0.690
CT	84 (42.2%)	43 (40.6%)	1.311 [0.664–2.588]	0.435
CC	25 (12.6%)	14 (13.2%)	0.961 [0.373–2.477]	0.935
CC + CT	109 (54.8%)	57 (53.8%)	1.207 [0.644–2.262]	0.558
Alleles				
T	264 (66.3%)	141 (66.5%)	-	-
C	134 (33.7%)	71 (33.5%)	1.059 [0.667–1.682]	0.807
Age ^b^			**1.127 [1.097–1.158]**	**<0.0001**
Sex				
Male	130 (65.3%)	83 (78.3%)	-	-
Female	69 (34.7%)	23 (21.7%)	**0.250 [0.118–0.529]**	**<0.001**
***HOTAIR* rs12826786**				
Genotype				
CC	94 (47.2%)	52 (49.1%)	-	0.196
CT	84 (42.2%)	48 (45.3%)	1.359 [0.704–2.624]	0.360
TT	21 (10.6%)	6 (5.7%)	0.439 [0.126–1.527]	0.196
TT + CT	105 (52.8%)	54 (50.9%)	1.136 [0.608–2.124]	0.689
Alleles				
C	272 (68.3%)	152 (71.7%)	-	-
T	126 (31.7%)	60 (28.3%)	0.913 [0.565–1.473]	0.708
Age ^b^			**1.128 [1.098–1.159]**	**<0.0001**
Sex				
Male	130 (65.3%)	83 (78.3%)	-	-
Female	69 (34.7%)	23 (21.7%)	**0.235 [0.110–0.503]**	**<0.001**

^a^ Odds ratio (OR) with 95% confidence intervals (CI) and ^b^ age as a continuous variable. Bold-faced values represent statistically significant differences (*p* < 0.05). The odds ratios and *p*-values for age and sex indicate those determined in multivariable logistic regression using the three groups of genotypes (CC, CT, and TT genotypes) individually.

**Table 4:** Multivariable Cox analyses of the association between the *HOTAIR* rs920778 and rs12826786 SNPs and overall survival in all bladder cancer patients, male patients, and patients with tumors staged as pT2.

Polymorphism	All Patients	HR [95% CI] ^a^	*p*- Value	Male Patients	HR [95% CI] ^a^	*p*- Value	pT2 Patients	HR [95% CI] ^a^	*p*-Value
***HOTAIR*** **rs920778**									
Genotype									
TT	49	-	0.322	38	-	0.086	8	-	0.111
CT	43	1.484 [0.783–2.812]	0.226	34	1.950 [0.936–4.062]	0.075	9	**4.248 [1.026–17.589]**	**0.046**
CC	14	1.924 [0.716–5.173]	0.195	11	2.841 [0.975–8.278]	0.056	3	4.497 [0.812–24.890]	0.085
CC+CT	57	1.550 [0.840–2.858]	0.161	45	**2.098 [1.047–4.204]**	**0.037**	12	**4.313 [1.099–16.931]**	**0.036**
Age at diagnosis ^b^		**1.051 [1.014–1.090]**	**0.007**		1.041 [0.999–1.083]	0.053		1.014 [0.962–1.069]	0.600
Sex				-	-	-			
Male	83	-	-	-	-	-	18	-	-
Female	23	0.838 [0.378–1.858]	0.664	-	-	-	2	1.271 [0.132–12.218]	0.835
Pathological stage (pT)							-	-	-
pTis	4	-	**<0.0001**	4	-	**<0.0001**	-	-	-
pTa	47	0.801 [0.102–6.285]	0.833	35	0.619 [0.077–5.001]	0.652	-	-	-
pT1	22	1.067 [0.128–8.918]	0.953	14	1.230 [0.144–10.541]	0.850	-	-	-
pT2	20	3.027 [0.391–23.414]	0.289	18	3.075 [0.394–23.982]	0.284	-	-	-
pT3	9	2.765 [0.318–24.022]	0.357	8	2.928 [0.320–26.790]	0.341	-	-	-
pT4	4	**40.661 [4.095–403.734]**	**0.002**	4	**39.680 [3.906–403.059]**	**0.002**	-	-	-
***HOTAIR*** **rs12826786**									
Genotype									
CC	52	-	0.198	41	-	**0.038**	7	-	**0.043**
CT	48	1.574 [0.837–2.960]	0.159	37	**2.332 [1.136–4.790]**	**0.021**	11	**6.343 [1.303–30.883]**	**0.022**
TT	6	2.547 [0.776–8.365]	0.123	5	3.384 [0.936–12.236]	0.063	2	**11.504 [1.366–96.901]**	**0.025**
TT+CT	54	1.644 [0.888–3.045]	0.114	42	**2.432 [1.207–4.898]**	**0.013**	13	**6.788 [1.427–32.284]**	**0.016**
Age at diagnosis ^b^		**1.051 [1.013–1.091]**	**0.008**		1.036 [0.995–1.078]	0.089		0.995 [0.938–1.055]	0.868
Sex				-	-	-			
Male	83	-	-	-	-	-	18	-	-
Female	23	0.815 [0.369–1.800]	0.612	-	-	-	2	0.437 [0.049–3.887]	0.458
Pathological stage (pT)							-	-	-
pTis	4	**-**	**<0.0001**	4	**-**	**<0.0001**	-	-	-
pTa	47	0.847 [0.108–6.625]	0.874	35	0.639 [0.079–5.180]	0.675	-	-	-
pT1	22	1.112 [0.133–9.330]	0.922	14	1.320 [0.154–11.270]	0.800	-	-	-
pT2	20	2.962 [0.385–22.799]	0.297	18	3.245 [0.419–25.134]	0.260	-	-	-
pT3	9	2.553 [0.289–22.565]	0.399	8	2.680 [0.285–25.204]	0.389	-	-	-
pT4	4	**41.616 [4.177–414.679]**	**0.001**	4	**38.968 [3.831–396.417]**	**0.002**	-	-	-

^a^ Hazard ratio (HR) with 95% confidence intervals (CI) and ^b^ age as a continuous variable. Bold-faced values represent statistically significant differences (*p* < 0.05). Hazard ratios and *p*-values for age at diagnosis, sex, and primary tumor pathological stages indicate those determined in multivariable Cox regression using the three groups of genotypes (CC, CT, and TT genotypes) individually.

## Data Availability

The data presented in this article are available upon request.

## Supplementary Materials

The following supporting information can be downloaded at: https://www.mdpi.com/article/10.3390/cancers16020434/s1, Table S1. Age and sex distribution in cancer-free controls and bladder cancer patients. Table S2. Univariable logistic regression analysis of the association between *HOTAIR* rs920778 and rs12826786 genetic variants and bladder cancer risk. Table S3. Multivariable logistic regression analysis of the association between *HOTAIR* rs920778 and rs12826786 genetic variants and bladder cancer risk. Table S4. Multivariable Cox analyses of the association between *HOTAIR* rs920778 and rs12826786 SNPs and overall survival in all bladder cancer patients with tumors staged as pT2, pT3 or pT4. Table S5. Multivariable Cox analyses of the association between *HOTAIR* rs920778 and rs12826786 SNPs and recurrence-free survival in all bladder cancer patients and in patients over 65 years.
