# Supplementary material for: Relevance of HOTAIR rs920778 and rs12826786 Genetic Variants in Bladder Cancer Risk and Survival
Source: Cancers (Basel). 2024 Jan 19;16(2):434. doi: 10.3390/cancers16020434 (PMC10814037; doi:10.3390/cancers16020434)
Supplement: Supplementary file 1 [file cancers-16-00434-s001.zip › cancers-2808201-supplementary.pdf]

cancers

MDPI

Article

Relevance of *HOTAIR* rs920778 and rs12826786 Genetic Variants in Bladder Cancer Risk and Survival

Eduarda P. Martins, Joana Vieira de Castro, Rita Fontes, Sara Monteiro-Reis, Rui Henrique, Carmen Jerónimo and Bruno M. Costa

Supplementary Materials

Supplementary Materials include:

- Supplementary tables:
  - **Table S1.** Age and sex distribution in cancer-free controls and bladder cancer patients.
  - **Table S2.** Univariable logistic regression analysis of the association between *HOTAIR* rs920778 and rs12826786 genetic variants and bladder cancer risk.
  - **Table S3.** Multivariable logistic regression analysis of the association between *HOTAIR* rs920778 and rs12826786 genetic variants and bladder cancer risk.
  - **Table S4.** Multivariable Cox analyses of the association between *HOTAIR* rs920778 and rs12826786 SNPs and overall survival in all bladder cancer patients with tumors staged as pT2, pT3 or pT4.
  - **Table S5.** Multivariable Cox analyses of the association between *HOTAIR* rs920778 and rs12826786 SNPs and recurrence-free survival in all bladder cancer patients and in patients over 65 years.

Table S1. Age and sex distribution in cancer-free controls and bladder cancer patients.

	**Controls**		**Cases**
	**All controls (n = 199)**	**Matched controls (n = 93)**	**All cases (n = 106)**
Age			
Range	27 – 85	45 – 85	37 – 91
Mean	46	59	67
Median	43	56	69
Sex			
Male	130	55	83
Female	69	38	23

Table S2. Univariable logistic regression analysis of the association between *HOTAIR* rs920778 and rs12826786 genetic variants and bladder cancer risk.

Polymorphism	Matched controls	Cases	OR [95% CI][a]	*p*-value
***HOTAIR* rs920778**				
Genotype				
TT	43 (46.2%)	49 (46.2%)	–	0.922
CT	36 (38.7%)	43 (40.6%)	1.048 [0.573 – 1.916]	0.878
CC	14 (15.1%)	14 (13.2%)	0.878 [0.376 – 2.046]	0.762
CC+CT	50 (53.8%)	57 (53.8%)	1.000 [0.572 – 1.749]	0.999
Alleles				
T	122 (65.6%)	141 (66.5%)	–	–
C	64 (34.4%)	71 (33.5%)	0.960 [0.633 – 1.455]	0.847
***HOTAIR* rs12826786**				
Genotype				
CC	44 (47.3%)	52 (49.1%)	–	0.427
CT	39 (41.9%)	48 (45.3%)	1.041 [0.581 – 1.865]	0.891
TT	10 (10.8%)	6 (5.7%)	0.508 [0.171 – 1.508]	0.222
TT+CT	49 (52.7%)	54 (50.9%)	0.932 [0.534 – 1.628]	0.806
Alleles				
C	127 (68.3%)	152 (71.7%)	–	–
T	59 (31.7%)	60 (28.3%)	0.850 [0.553 – 1.306]	0.458

[a]Odds ratio (OR) with 95% confidence intervals (CI).

Table S3. Multivariable logistic regression analysis of the association between *HOTAIR* rs920778 and rs12826786 genetic variants and bladder cancer risk.

Polymorphism	Matched controls	Cases	OR [95% CI][a]	*p*-value
***HOTAIR* rs920778**				
Genotype				
TT	43 (46.2%)	49 (46.2%)	–	0.663
CT	36 (38.7%)	43 (40.6%)	1.296 [0.654 – 2.568]	0.457
CC	14 (15.1%)	14 (13.2%)	0.892 [0.352 – 2.258]	0.809
CC+CT	50 (53.8%)	57 (53.8%)	1.168 [0.623 – 2.187]	0.629
Alleles				
T	122 (65.6%)	141 (66.5%)	–	–
C	64 (34.4%)	71 (33.5%)	1.019 [0.641 – 1.620]	0.935
Age[b]			**1.088 [1.055 – 1.122]**	**< 0.0001**
Sex				
Male	55 (59.1%)	83 (78.3%)	–	–
Female	38 (40.9%)	23 (21.7%)	**0.273 [0.134 – 0.556]**	**< 0.001**
***HOTAIR* rs12826786**				
Genotype				
CC	44 (47.3%)	52 (49.1%)	–	0.198
CT	39 (41.9%)	48 (45.3%)	1.320 [0.681 – 2.561]	0.411
TT	10 (10.8%)	6 (5.7%)	0.435 [0.130 – 1.454]	0.176
TT+CT	49 (52.7%)	54 (50.9%)	1.099 [0.588 – 2.056]	0.767
Alleles				
C	127 (68.3%)	152 (71.7%)	–	–
T	59 (31.7%)	60 (28.3%)	0.892[0.552 – 1.440]	0.640
Age[b]			**1.089 [1.055 – 1.123]**	**< 0.0001**
Sex				
Male	55 (59.1%)	83 (78.3%)	–	–
Female	38 (40.9%)	23 (21.7%)	**0.256 [0.124 – 0.529]**	**< 0.001**

[a]Odds ratio (OR) with 95% confidence intervals (CI); [b]age as continuous variable. Bold-faced values represent statistically significant differences ($p < 0.05$). Odds ratios and *p*-values for age at diagnosis and sex indicate those determined in multivariable logistic regression using the three groups of genotypes (CC, CT, and TT genotypes) individually.

Table S4. Multivariable Cox analyses of the association between *HOTAIR* rs920778 and rs12826786 SNPs and overall survival in all bladder cancer patients with tumors staged as pT2, pT3 or pT4.

**Polymorphism**	**pT2-pT4 patients**	**HR [95% CI][a]**	***p*-value**
***HOTAIR* rs920778**			
Genotype			
TT	15	–	0.359
CT	13	1.901 [0.742 – 4.868]	0.181
CC	5	1.800 [0.584 – 5.545]	0.306
CC+CT	18	1.865 [0.793 – 4.386]	0.153
Age at diagnosis[b]		1.010 [0.965 – 1.056]	0.679
Sex			
Male	30	–	–
Female	3	0.419 [0.083 – 2.113]	0.292
***HOTAIR* rs12826786**			
Genotype			
CC	13	–	0.119
CT	16	**2.865 [1.026 – 7.999]**	**0.044**
TT	4	2.494 [0.700 – 8.889]	0.159
TT+CT	20	**2.746 [1.046 – 7.210]**	**0.040**
Age at diagnosis[b]		1.001 [0.954 – 1.051]	0.962
Sex			
Male	30	–	–
Female	3	0.300 [0.057 – 1.588]	0.157

[a]Hazard ratio (HR) with 95% confidence intervals (CI); [b]age as continuous variable. Bold-faced values represent statistically significant differences ($P < 0.05$). Hazard ratios and *p*-values for age at diagnosis, sex, and primary tumor pathological stage indicate those determined in multivariable Cox regression using the three groups of genotypes (CC, CT, and TT genotypes) individually.

Table S5. Multivariable Cox analyses of the association between *HOTAIR* rs920778 and rs12826786 SNPs and recurrence-free survival in all bladder cancer patients and in patients over 65 years.

Polymorphism	All patients	HR [95% CI][a]	*p*-value	≥ 65 years patients	HR [95% CI][a]	*p*-value
***HOTAIR* rs920778**						
Genotype						
TT	49	–	0.951	34	–	0.343
CT	43	1.052 [0.621 – 1.783]	0.850	25	1.633 [0.832 – 3.206]	0.154
CC	14	1.131 [0.505 – 2.535]	0.764	8	1.091 [0.346 – 3.439]	0.882
CC+CT	57	1.069 [0.650 – 1.756]	0.793	33	1.515 [0.787 – 2.914]	0.213
Age at diagnosis[b]		0.996 [0.969 – 1.024]	0.788	–	–	–
Sex						
Male	83	–	–	48	–	–
Female	23	0.983 [0.525 – 1.841]	0.957	19	1.068 [0.528 – 2.160]	0.854
Pathological stage (pT)						
pTis	4	–	**0.004**	3	–	**0.005**
pTa	47	0.842 [0.197 – 3.599]	0.816	27	1.504 [0.193 – 11.700]	0.697
pT1	22	0.915 [0.203 – 4.130]	0.908	17	1.625 [0.195 – 13.510]	0.653
pT2	20	1.534 [0.346 – 6.797]	0.573	13	1.791 [0.211 – 15.178]	0.593
pT3	9	0.811 [0.159 – 4.128]	0.801	5	1.029 [0.104 – 10.216]	0.980
pT4	4	**7.475 [1.326 – 42.147]**	**0.023**	2	**96.947 [5.691 – 1651.514]**	**0.002**
***HOTAIR* rs12826786**						
Genotype						
CC	52	–	0.211	36	–	**0.025**
CT	48	1.298 [0.781 – 2.155]	0.314	28	1.916 [0.998 – 3.678]	0.051
TT	6	2.223 [0.870 – 5.680]	0.095	3	**4.985 [1.320 – 18.829]**	**0.018**
TT+CT	54	1.386 [0.851 – 2.258]	0.190	31	**2.057 [1.091 – 3.877]**	**0.026**
Age at diagnosis[b]		0.999 [0.972 – 1.026]	0.927	–	–	–
Sex						
Male	83	–	–	48	–	–
Female	23	0.961 [0.512 – 1.807]	0.902	19	0.948 [0.472 – 1.907]	0.882
Pathological stage (pT)						
pTis	4	–	**0.004**	3	–	**0.003**
pTa	47	0.857 [0.201 – 3.660]	0.835	27	1.590 [0.205 – 12.341]	0.657
pT1	22	0.945 [0.209 – 4.283]	0.942	17	1.862 [0.225 – 15.447]	0.564
pT2	20	1.492 [0.334 – 6.657]	0.600	13	1.591 [0.191 – 13.223]	0.668
pT3	9	0.709 [0.137 – 3.678]	0.683	5	0.945 [0.095 – 9.423]	0.961
pT4	4	**7.526 [1.327 – 42.677]**	**0.023**	2	**116.809 [6.814 – 2002.337]**	**0.001**

[a]Hazard ratio (HR) with 95% confidence intervals (CI); [b]age as continuous variable. Bold-faced values represent statistically significant differences ($p < 0.05$). Hazard ratios and *p*-values for age at diagnosis, sex, and primary tumor pathological stage indicate those determined in multivariable Cox regression using the three groups of genotypes (CC, CT, and TT genotypes) individually.
